# Comprehensive Evaluation of Mung Bean Germplasm Resources Based on DUS Test Characteristics

**DOI:** 10.3390/plants15060932

**Published:** 2026-03-18

**Authors:** Leyong Feng, Juanjuan Ma, Jin Yu, Jianhong Ren, Xiongfei Jiao

**Affiliations:** 1 Maize Research Institute, Shanxi Agricultural University, Xinzhou 034000, China; ymsfly@163.com (L.F.); xzdusyj7017@163.com (J.Y.); 2 College of Agronomy, Shanxi Agricultural University, Taiyuan 030043, China; sxndmajj@163.com; 3 College of Life Sciences, Shanxi Agricultural University, Jinzhong 030800, China; renjh@sxau.edu.cn

**Keywords:** mung bean, DUS test characteristics, cluster analysis, linear regression analysis, breeding

## Abstract

The Distinctness, Uniformity, and Stability (DUS) testing guidelines for mung beans (*Vigna radiata* L.) offer a standardized framework for new variety assessment. Although these guidelines are essential for variety management, the actual efficiency and breeding value of the 31 specified DUS characteristics in improving yield potential remain largely underexplored and lack systematic validation. To address this critical research gap, 180 genetically diverse mung bean accessions were analyzed using principal component analysis (PCA) and correlation analysis. The results revealed intrinsic relationships among characteristics and identified key variation dimensions centered on “plant morphology”, “pod characteristics”, and “seed characteristics”. Cluster analysis classified the 180 accessions into four distinct clusters. Cluster 2, in particular, offers a clear selection reference for breeding materials targeting high-yield and quality. The DTOPSIS (Dynamic Technique for Order Preference by Similarity to Ideal Solution) multi-criteria decision-making model was applied, with index weights assigned using an objective weighting method. Following systematic evaluation, Yingge 2 was identified as an outstanding phenotype. Breeders may refer to its quantitative characteristics in subsequent breeding cycles. Linear regression analysis was employed to construct a yield prediction model, identifying leaf greenness, pod number per plant, and hundred-grain weight as three core DUS characteristics with statistically significant effects on final yield (*p* < 0.05). This study performed a systematic, multi-dimensional analysis and comprehensive evaluation of mung bean germplasm resources based on DUS characteristics, with the aim of identifying key yield-related DUS traits, screen elite germplasm for high-yield breeding, and providing a theoretical basis and practical reference for the efficient improvement and selective breeding of new mung bean varieties.

## 1. Introduction

Mung bean (*Vigna radiata* L.) is an important short-duration leguminous crop that can grow in tropical, subtropical, and some temperate regions [1]. Mung bean is rich in easily digestible protein, carbohydrates, amino acids, essential micronutrients and natural antioxidants, exhibiting exceptional nutritional value. It also has medicinal properties and contributes to sustainable agricultural development via biological nitrogen fixation, which helps optimize cropping systems and boost the high-quality seed industry. Accordingly, mung bean has gained widespread attention across the globe [2,3,4]. As one of the centers of origin of mung bean, China has a cultivation history of more than 2000 years, giving the crop great economic and cultural significance [5]. However, under increasingly complex climate change and evolving market demands, the mung bean industry faces severe challenges in yield enhancement, stable nutritional and edible quality, and breeding varieties with broad-spectrum stress resistance [6].

Cultivating and protecting improved varieties is central to addressing these challenges. Plant variety protection relies on Distinctness, Uniformity, and Stability (DUS) testing, which serves as a scientifically rigorous evaluation system for new varieties [7]. DUS testing is simple to analyze, cost-effective, and does not rely on experimental technologies, playing a crucial role in the production and certification of high-quality seeds [8]. DUS testing has been successfully applied in breeding programs for major crops such as maize and rice, providing effective support for variety identification, protection of breeders’ rights, and guiding selection efforts. Applying it to mung bean breeding can similarly offer a reliable framework for germplasm identification and screening of desirable characteristics [9,10].

Despite its considerable potential, the systematic application of DUS testing in mung bean remains insufficient. Current studies are often limited to small-scale germplasm or single ecological regions, making it difficult to comprehensively reveal the population variation patterns of target characteristics [11]. Furthermore, the current description of characteristics falls short in covering key characteristics, particularly yield components, and lacks systematic correlation with yield performance. This in turn constrains its predictive and applied value in breeding [12]. Additionally, the loss of local mung bean germplasm resources is a widespread global concern, highlighting an urgent need for comprehensive and systematic characterization to facilitate the identification and conservation of elite genetic resources [13].

To bridge this knowledge gap, current research on the distinctness, uniformity and stability (DUS) testing of mung bean remains insufficient. The identification of qualitative and quantitative traits closely associated with yield, quality and adaptability is largely inadequate. Furthermore, most existing studies are restricted to small sample sizes and lack large-scale systematic investigations, which limits the comprehensive understanding of genetic diversity and trait variation patterns in mung bean germplasm. Therefore, this study conducted a systematic DUS-based evaluation of 180 mung bean varieties, integrating characteristics analysis with yield assessment. We aimed to analyze the correlation between DUS traits and yield, identify elite varieties with desirable traits and high-yield potential, and provide a scientific basis for the genetic improvement and yield enhancement of mung bean. 

## 2. Results

### 2.1. Phenotypic Observation and Characteristic Analysis in DUS Testing

**Figure 1:**
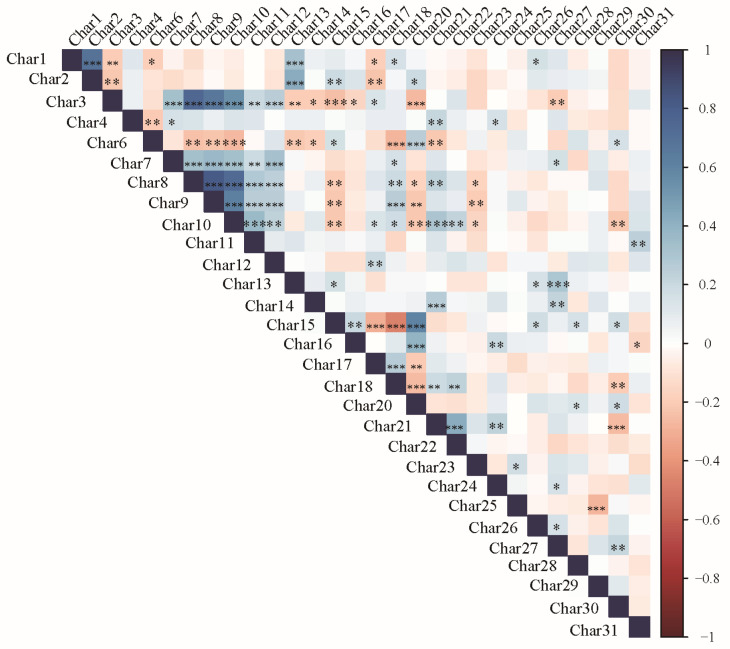
Correlation analysis and heatmap visualization of 29 DUS test characteristics in 180 mung bean germplasm accessions. The heatmap visualizes pairwise correlation coefficients among the 29 DUS test characteristics. The color gradient represents the strength and direction of correlations: dark blue indicates a strong positive correlation (coefficient near 1), white represents no correlation (coefficient near 0), and dark red denotes a strong negative correlation (coefficient near −1). Asterisks indicate the significance level of correlations: * *p* < 0.05, ** *p* < 0.01, *** *p* < 0.001.

A systematic observation and analysis of 31 phenotypic characteristics was performed on 180 mung bean germplasms. The results indicated that different characteristics exhibited distinct frequency distributions (Figure 1). Among these germplasms, only one phenotypic type was observed for characteristic 5 and characteristic 19; due to their lack of diversity, these two characteristics were excluded from further analysis. Characteristics 1, 6, 8, 11, 12, 24, 25, 26 and 31 each displayed two phenotypic types. Within this group, characteristics 11, 22 and 25 showed relatively narrow variation ranges, each presenting a single-grade distribution. In contrast, characteristics 13, 21 and 28 exhibited the widest distribution ranges, covering nine phenotypic grades. To comprehensively characterize the phenotypic variation, Table 1 summarizes the mean, standard deviation, coefficient of variation (CV), and Shannon–Weaver diversity index (H′) for all 31 characteristics. A higher CV reflects greater variability among observations. The analysis revealed that characteristics 1, 8, 12 and 29 displayed relatively high variation, with CVs of 0.510, 0.529, 0.775 and 1.278, respectively. In comparison, characteristics 13, 22 and 25 showed low variation, with corresponding CVs of 0.025, 0.094 and 0.063. The Shannon–Weaver diversity index was used to assess the evenness of phenotypic distribution across germplasms, with higher values indicating more balanced characteristic representation. The diversity indices of characteristics 2, 13, 15, 18 and 21 all exceeded 1.5. Among these, characteristic 18 had the highest index (2.058), while characteristic 25 showed the lowest (0.034).

### 2.2. Correlation Among Phenotypic Characteristics

Correlation analyses were performed across 29 characteristics, and the resulting relationships were visualized in a heatmap (Figure 1). The heatmap employs a color gradient to intuitively depict the strength and direction of correlations among the characteristics: dark blue represents a strong positive correlation (correlation coefficient near 1), white indicates no correlation (correlation coefficient near 0), and dark red denotes a strong negative correlation (correlation coefficient near −1).

The analysis revealed a very strong positive correlation between characteristic 1 and characteristic 2 (correlation coefficient close to 1), while characteristic 15 showed a significant negative correlation with characteristic 18 (correlation coefficient close to −1), indicating an opposing trend in their variation. Further examination demonstrated that the number of significant correlations varied across characteristics. Characteristic 3 exhibited the highest number of significant correlations, showing strong positive correlations with characteristics 7, 8, 9, 10, 11, 12, and 16, and strong negative correlations with characteristics 13, 14, 15, and 17. In contrast, characteristic 29 displayed only one significant correlation—a negative correlation with characteristic 25—representing the fewest significant correlations among all characteristics examined.

### 2.3. Clustering Analysis

**Figure 2:**
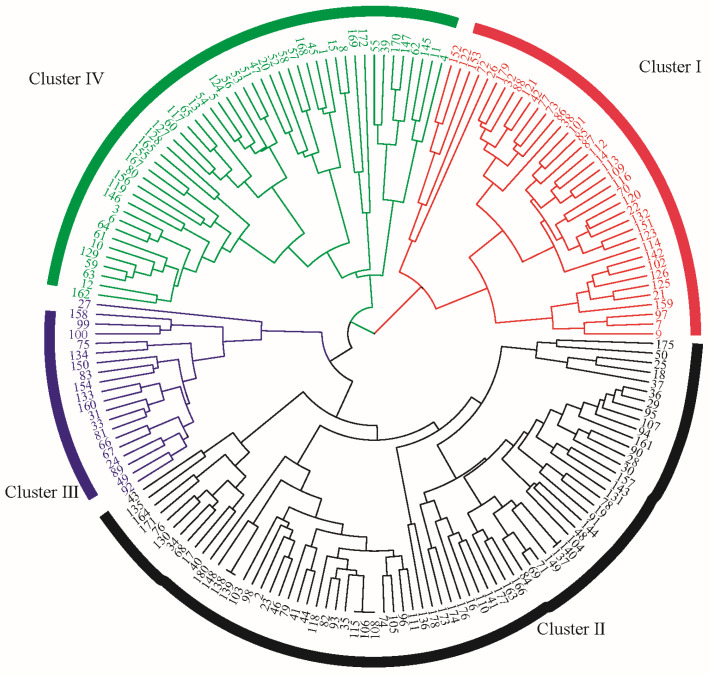
Cluster analysis of 180 mung bean germplasm accessions based on 29 key phenotypic characteristics.

A cluster analysis was performed on 29 key phenotypic characteristics. The data were first standardized, and a hierarchical clustering approach was applied for further examination. As illustrated in Figure 2, when the Euclidean distance was set to 10, the 180 accessions were grouped into four distinct clusters. The mean values of the characteristics for each group are provided in Table 2. Cluster 1 comprises 38 varieties. Within this cluster, all accessions share a code of 1 for characteristic 1. Additionally, this cluster shows the lowest mean values for characteristics 3, 4, 7, 8, 9, 10, 11, 12, 14, 21, 28, 29, and 31 compared to the other clusters. Since most of these characteristics are related to color, the results suggest that accessions in Cluster 1 generally exhibit lighter coloration across various plant parts. Cluster 2 contains the largest number of varieties (73). It displays the highest mean values for characteristics 4, 7, 8, 9, 14, 18, 21, and 27. These characteristics predominantly reflect pre-flowering color characteristics as well as several morphological measurements. This pattern indicates that varieties in Cluster 2 tend to have darker leaves and are characterized by longer pods, a higher number of pods per plant, and greater hundred-grain weight. Given the close association of these characteristics with yield, Cluster 2 accessions are likely to exhibit superior yield performance. Cluster 3 includes the fewest varieties (23). It recorded the highest values for characteristics 10, 12, 15, 20, 28, 30, and 31, but the lowest values for characteristics 17, 18, 24, and 26. This profile suggests that accessions in this cluster possess relatively tall plant stature with more compact branching, yet bear a lower number of pods. Cluster 4 consists of 49 varieties. It shows the highest codes for characteristics 2, 13, 17, 22, and 26, and the lowest codes for characteristics 23 and 27. These results imply that accessions in Cluster 4 have a longer growth cycle but a smaller hundred-grain weight.

### 2.4. Principl Component Analysis

**Figure 3:**
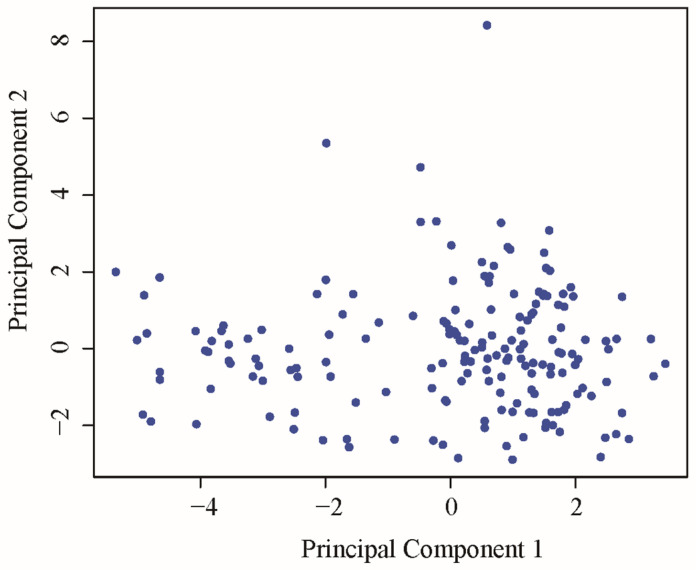
PCA scatter plot of 180 mung bean germplasm resources based on the first two principal components.

Principal component analysis (PCA) was performed to identify the main characteristics among 180 mung bean germplasm resources. As presented in Table 3, ten of the 31 eigenvalues exceeded 1, with a cumulative contribution rate of 66.49%, indicating that these ten principal components accounted for the majority of the variation in the 31 DUS test characteristics. PC1 was mainly associated with leaf color attributes, PC2 was primarily related to plant morphology, PC3 reflected petiole characteristics, PC4 corresponded to seed-related traits, and PC5 represented main stem attributes. The remaining principal components were associated with pod color, pod shape, branch number, seed coat luster, and stem pubescence. Furthermore, as shown in Figure 3, the PCA scatter plot based on the first two principal components revealed that all accessions were continuously distributed without distinct independent clusters, suggesting abundant genetic variation present in the tested mung bean germplasm resources.

### 2.5. Comprehensive Evaluation of Agronomic Characteristics in DUS Testing

By substituting the standardized values of the 29 DUS test characteristics (excluding characteristics 5 and 19) into the ten principal components, the scoring formulas for each component can be derived:

(1) $${FAC}_{i}=\sum_{k=1}^{q}c_{ik}x_{k}$$

The index *i* ranges from 1 to the total number of components (here, 10), while *k* varies from 1 to the number of characteristics (here, 29). Based on the values of FAC1 through FAC10, the DTOPSIS method was applied to compute U1 to U10. The weights were assigned according to the contribution rates of each principal component, and a comprehensive evaluation formula D (representing the composite evaluation score) for different mung bean varieties was established through principal component analysis as follows:

D = 0.259 × U1 + 0.141 × U2 + 0.118 × U3 + 0.083 × U4 + 0.077 × U5 + 0.073 × U6 + 0.068 × U7 + 0.064 × U8 + 0.060 × U9 + 0.056 × U10(2)

Using this model, the composite scores of 180 tested varieties were calculated (Table 4). A higher D value indicates better overall phenotypic performance. The mean comprehensive evaluation score across all 180 mung bean accessions was 0.5398. The variety numbered 15 achieved the highest composite score (0.7093), which was primarily characterized by darker pigmentation in plant tissues, a greater number of branches, higher pod and seed counts, and increased yield.

### 2.6. Linear Regression Analysis

**Figure 4:**
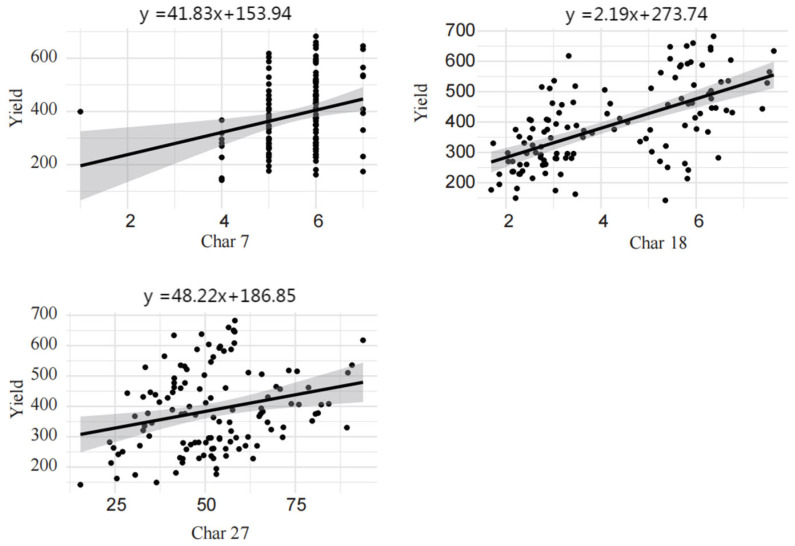
Linear regression analysis of yield in relation to its associated characteristics. The solid line in the figure represents the fitted linear regression model, and the shaded area indicates the 95% confidence interval.

A correlation analysis between yield and all DUS characteristics was performed (Table 5), revealing that only characteristics 7, 18, and 27 showed significant correlations with yield. Subsequently, a linear regression model was fitted with yield as the dependent variable and characteristics 7, 18, and 27 as independent variables. The results are presented in Figure 4. The regression analysis indicated a significant positive linear relationship between mung bean yield and each of the three characteristics. Specifically, the equation for characteristic 7 was y = 41.83x + 153.94 (R^2^ = 0.07, *p* < 0.05), suggesting that yield increases steadily with increasing characteristic 7 values. Each unit increase in characteristic 7 was associated with an average yield increase of 41.83 units, starting from a baseline yield of 153.94 units. These results imply that characteristic 7 contributes moderately to mung bean yield and may be an important factor influencing yield variation. Maintenance of elevated leaf greenness throughout the growing season prolongs leaf functional longevity, reduces premature senescence, and promotes the partitioning of dry matter to grains, which in turn underpins the enhancement of grain yield in mung bean. For characteristic 18, the fitted equation was y = 2.19x + 273.74 (R^2^ = 0.07, *p* < 0.05). Although the regression slope was relatively small, it still reflected a positive association between characteristic 18 and mung bean yield. Each unit increase in characteristic 18 corresponded to an average yield increase of 2.19 units, with an intercept of 273.74 units. Thus, while characteristic 18 also exhibited a positive effect on yield, its influence slightly weaker appeared weaker compared to characteristic 7. Characteristic 27 showed a more pronounced relationship with mung bean yield, following the equation y = 48.22x + 186.85 (R^2^ = 0.37, *p* < 0.05). Here, each unit increase in characteristic 27 was associated with an average yield increase of 48.22 units, indicating a stronger strong positive effect. These findings suggest that characteristic 27 may play a critical role in determining mung bean yield. Pod number per plant and 100-seed weight are the core agronomic traits governing mung bean yield. In production practice, the optimal coordination of these two traits can be realized through the screening and breeding of adapted varieties, which provides a theoretical foundation and practical guidance for optimizing high-yield cultivation techniques and advancing high-yield and high-quality breeding of mung beans.

## 3. Discussion

This study identified three core yield-predictive traits (Characteristic 7: Leaf: Degree of light green color, Characteristic 18: Plants: Number of pods, Characteristic 27: Seed: Weight per Hundred Seeds) with significant positive correlations with mung bean yield through correlation and linear regression analysis, among which Characteristic 27 showed the strongest correlation (r = 0.66, *p* < 0.001), followed by Characteristic 7 and 18 (both r = 0.26, *p* < 0.05). Linear regression models further quantified the yield contribution of these traits: each unit increase in Characteristics 7, 18 and 27 was associated with an average yield increase of 41.83, 2.19 and 48.22 units, respectively, indicating that these three traits are the key phenotypic determinants of mung bean yield formation and can serve as reliable yield-predictive indicators in DUS testing and breeding practice. On the basis of this core finding, we further discussed the phenotypic variation characteristics, trait correlation patterns, germplasm clustering characteristics and comprehensive evaluation system of mung bean DUS traits, and explored the genetic and breeding implications of these results for mung bean genetic improvement. In addition, this study found that Characteristic 5 (Leaves: Number of small leaves) and Characteristic 19 (Plants: Pod-frying property) each displayed only a single phenotypic state, which suggests that these characteristics may be under high genetic constraint [14]. From an evolutionary perspective, such phenotypic conservation likely reflects the essential role of these characteristics in legume adaptation. For instance, leaflet number is likely under stringent control by developmental stability genes, such as those in the KNOX family [15]. Similarly, the absence of explosive pod shattering may be closely linked to directional selection for pod indehiscence during artificial domestication [16,17]. In contrast, the four highly variable characteristics (characteristics 1, 8, 12, and 29) exhibited distinct genetic profiles. Their high coefficients of variation (0.510–1.278) indicate not only polygenic regulation but also considerable phenotypic plasticity, which may facilitate environmental adaptation. Notably, from a population genetics standpoint, the high diversity index of characteristic 18 (pod number) (H′ = 2.058) suggests that this characteristic has retained substantial allelic variation throughout natural and artificial selection [18]. This diversity provides a valuable genetic foundation for further exploring yield potential via hybridization breeding [19].

Correlation analysis across 29 characteristics indicated a particularly noteworthy polygenic nature for characteristic 3. Its broad associations with 13 other characteristics suggest that this characteristic may influence multiple developmental processes, potentially mediated by hormone signaling pathways or transcription factor cascades [20,21]. From a breeding standpoint, the positive correlation between characteristic 7 (leaf greenness) and characteristic 18 (pod number) supports the applicability of the “source–sink” theory [22]. In contrast, the negative correlation between characteristic 15 (plant height) and characteristic 18 highlights key trade-offs to be balanced in plant architecture improvement [23,24].

The clustering analysis based on phenotypic characteristics not only enabled the systematic classification of germplasm resources, but also suggested specific breeding strategies for different improvement objectives. The low anthocyanin content observed in Cluster 1 may be associated with its ecological adaptability, offering candidate materials for breeding varieties suited to specific ecological regions. Group 2 exhibited favorable yield performance. The plant architecture characteristics of Cluster 3 indicated potential for yield enhancement through morphological improvement, although its weak reproductive growth also implied the necessity for hybrid-based breeding to balance vegetative and reproductive development. Despite the disadvantage of low hundred-grain weight, Cluster 4 showed valuable characteristics such as a long growth period, which could be useful in selecting late-maturing cultivars. This phenotype-based classification system provides a scientific foundation for precise parental selection and targeted breeding programs.

Principal component analysis (PCA) effectively reduced the complexity of the characteristic system to ten principal components, which is methodologically valuable. The cumulative contribution rate of the first ten components reached 66.493%, slightly lower than the 70% commonly reported in other crop studies. This outcome, however, plausibly reflects the greater complexity of the genetic background in mung beans. Notably, the first three principal components predominantly represent leaf color, plant architecture, and seed characteristics—characteristics that are also of major interest in soybean breeding. From the perspective of optimizing test protocols, this dimensionality-reduction approach could substantially enhance the efficiency of Distinctness, Uniformity, and Stability (DUS) testing. By focusing on characteristics with higher loadings in the leading principal components, the workload can be significantly reduced without compromising identification accuracy. Moreover, the dominant representation of pod- and seed-related characteristics in the principal components has been statistically confirmed, underscoring their central role in yield formation.

The establishment of the DTOPSIS comprehensive evaluation system marks a significant advancement in germplasm resource assessment methodologies. By incorporating weighted evaluations across multiple characteristics, this approach effectively mitigates the limitations inherent in conventional single-characteristic screening. The determination of an overall mean score of 0.5398 provides an objective benchmark for evaluating varietal quality. The superior performance of variety No. 15, with a score of 0.7093, further validates the practical efficacy of this system. From a breeding application perspective, this comprehensive evaluation method not only enables the rapid identification of varieties with favorable overall characteristics but also facilitates the discovery of innovative materials characterized by distinctive characteristic combinations. Three key characteristics—leaf greenness, pod number, and hundred-grain weight—collectively explain 76% of the observed yield variation. From a quantitative genetics standpoint, this result underscores the central role of these characteristics in yield formation. Notably, the mechanisms through which these characteristics contribute to yield differ significantly: hundred-grain weight primarily enhances yield by increasing individual seed weight, pod number ensures yield stability through the multiplication of yield components, and leaf greenness provides the material basis for yield by improving photosynthetic efficiency. This collaborative yet distinct mode of characteristic contribution suggests that differentiated selection strategies should be adopted in breeding programs. For hundred-grain weight, direct selection is warranted; for pod number, coordination with plant architecture should be considered; and for leaf greenness, stability under varying environmental interactions merits attention. The development of this model lays the groundwork for predicting yield potential based on DUS (Distinctness, Uniformity, Stability) characteristics during early breeding stages, which is expected to substantially enhance breeding efficiency.

## 4. Materials and Methods

### 4.1. Plant Materials and Experiments Design

In this study, a total of 180 mung bean accessions were collected, comprising 134 landrace varieties and 46 breeding lines (Table S1). Field experiments were conducted in 2023 and 2024 at an experimental site located in Xinzhou City, Shanxi Province, China (geographical coordinates: 120°52′ E, 30°40′ N; elevation 791 m). A randomized complete block design was employed with three replications to ensure the reliability of the results. Sowing was carried out in mid- to late May each year. Each experimental plot measured 5 m in length and 2.4 m in width, containing six rows with a row spacing of 40 cm and a plant spacing of 15 cm. The plot size was designed to accommodate at least 120 plants per plot. All trials were established on sandy loam soil.

### 4.2. Measurement of Phenotypic Characteristics and Collection of Data

The characteristic assessments were conducted in accordance with the industry standard NY/T 2350-2013 [25]. A total of 31 characteristics were recorded (Table 6), comprising 3 qualitative characteristics (QL), 10 semi-qualitative (pseudo-qualitative) characteristics (PQ), and 18 quantitative characteristics (QN). Upon maturation, plants from three plots were sampled for seed evaluation. The standard per-acre yield of mung beans was calculated using established scientific methodologies.

### 4.3. Genetic Diversity Analysis

For visual characteristics, codes recorded over the two-year period were sequentially calibrated against the standards of the reference varieties as prescribed in the guidelines [25]. For measured characteristics, the mean values derived from 40 typical plants selected each year were used for statistical analysis [26,27]. Based on the mean (X) and standard deviation (s) of each quantitative characteristic, the 180 mung bean germplasm accessions were classified into nine grades using the following criteria: grade 1 for values < X − 2s, grade 10 for values > X + 2s, and intermediate grades assigned accordingly [28,29]. Genetic diversity for each characteristic was assessed using the Shannon diversity index (H′), calculated as H′ = −ΣPi ln Pi, where Pi denotes the frequency of the i-th phenotypic class and ln represents the natural logarithm [30,31].

### 4.4. Comprehensive Evaluation of Genetic Quality

The mung bean germplasm was evaluated using the Decision-making Trial and Evaluation Laboratory and Technique for Order Preference by Similarity to Ideal Solution (DTOPSIS) method [32,33]. The calculation formulas are as follows: FAC denotes distinct principal component groups. Within the i-th principal component group, FACi− and FACi+ correspond to the minimum and maximum values, respectively. The j-th value in the standardized dataset of the i-th principal component group is represented by Fij, while Uij denotes the j-th value in the i-th group. Subsequently, the contribution rates derived from the principal component analysis were incorporated into the comprehensive evaluation score formula for computation [34,35].

### 4.5. Statistical Analysis of the Data Was Performed

All statistical analyses were performed with IBM SPSS Statistics 22.0 (IBM Corp., Armonk, NY, USA) and R software (version 4.3.1, R Foundation for Statistical Computing, Vienna, Austria). Correlation, principal component, regression, and cluster analyses were performed using SPSS 22.0 software [36]. For the cluster analysis, the data were standardized prior to analysis, and clustering was carried out based on Euclidean distance with the Ward (sum of squared differences) method [37]. Segmented linear regression analysis and scatter plot visualization were carried out in R with the SiZer [38] and ggplot2 packages. All analyses were performed in triplicate to ensure repeatability and reliability. Data sorting, collation, and interspecific variation analysis were processed using Microsoft Excel 2021 (Microsoft Corp., Redmond, WA, USA) [39].

## 5. Conclusions

This study systematically analyzed 31 distinctness, uniformity, and stability (DUS) test characteristics across 180 mung bean germplasm resources using multiple statistical methods. The main findings are as follows: Correlation analysis revealed shared regulatory patterns among color-related characteristics. Cluster analysis categorized the germplasm into four groups, with Group 2 (73 accessions) exhibiting darker leaf color, longer pods, more large pods, and higher hundred-grain weight, offering clear selection guidance for high-yield and high-quality breeding. Principal component analysis simplified the characteristic system by extracting 10 principal components that accounted for 66.493% of the cumulative variance, highlighting leaf color, plant morphology, and seed characteristics as core sources of variation. The DTOPSIS comprehensive evaluation objectively ranked germplasm quality and identified ‘Yingge 2’ as having superior overall characteristics, with outstanding regional adaptability; breeders may thus consider prioritizing its quantitative characteristics in subsequent breeding cycles. Linear regression analysis quantified the contributions of leaf greenness, pod number, and hundred-grain weight to yield, yielding a predictive model that explained 76% of yield variation and providing a reliable tool for early selection. Together, these results improved the understanding of the genetic architecture underlying mung bean DUS characteristics and suggested that breeders could select for superior traits such as leaf greenness, pod number per plant, and 100-seed weight to enhance yield stability and productivity in mung bean.

## Figures and Tables

**Table 1:** Statistical analysis of 31 DUS test characteristics based on 180 mung bean germplasm accessions.

Characteristics	Max	Min	Mean	SD	CV	H′
Char 1	9	1	6.911	3.524	0.510	0.574
Char 2	54	27	36.283	4.898	0.135	1.807
Char 3	3	1	1.806	0.580	0.321	0.865
Char 4	3	1	2.456	0.582	0.237	0.843
Char 5	1	1	1.000	0.000	0.000	0.000
Char 6	3	2	2.594	0.492	0.190	0.675
Char 7	9	1	5.667	0.872	0.154	1.185
Char 8	9	1	6.783	3.585	0.529	0.591
Char 9	3	1	1.844	0.615	0.333	0.919
Char 10	3	1	2.522	0.835	0.331	0.667
Char 11	9	1	8.644	1.653	0.191	0.182
Char 12	9	1	2.339	2.988	1.278	0.451
Char 13	84	62	69.128	4.370	0.063	1.709
Char 14	3	1	1.050	0.265	0.252	0.188
Char 15	124.64	41.7	64.647	12.774	0.198	1.546
Char 16	10.4	1.9	4.183	1.025	0.245	1.011
Char 17	5	2	3.767	0.763	0.203	1.098
Char 18	124.65	7.8	57.758	22.110	0.383	2.058
Char 19	1	1	1.000	0.000	0.000	0.000
Char 20	13.45	5.15	7.728	1.240	0.160	0.962
Char 21	15.625	6.54	10.429	1.270	0.122	1.581
Char 22	15.5	6.667	11.919	1.121	0.094	0.199
Char 23	3	1	1.917	0.394	0.205	0.371
Char 24	2	1	1.256	0.437	0.348	0.568
Char 25	3	2	2.994	0.075	0.025	0.034
Char 26	2	1	1.878	0.328	0.175	0.371
Char 27	8.066	1.293	4.652	1.791	0.385	1.238
Char 28	3	1	2.528	0.522	0.207	0.742
Char 29	5	1	1.300	1.008	0.775	0.389
Char 30	3	1	2.186	0.625	0.286	0.937
Char 31	9	1	7.994	2.644	0.331	0.382

Note: standard deviation (SD); coefficient of variation (CV); Shannon diversity index (H′).

**Table 2:** Average values of DUS test characteristics in different clusters of 180 mung bean germplasm accessions.

Characteristics	Clusters				Characteristics	Clusters			
	I	II	III	IV		I	II	III	IV
Char 1	1.00	9.00	9.00	7.53	Char 17	3.71	3.75	3.65	3.88
Char 2	35.29	36.04	37.00	37.12	Char 18	57.72	60.20	47.26	58.44
Char 3	1.18	2.10	2.10	1.73	Char 19	1.00	1.00	1.00	1.00
Char 4	2.39	2.55	2.35	2.41	Char 20	8.02	7.49	8.21	7.66
Char 5	1.00	1.00	1.00	1.00	Char 21	9.99	10.71	10.11	10.49
Char 6	2.76	2.53	2.60	2.55	Char 22	11.39	12.07	11.84	12.13
Char 7	5.26	5.90	5.60	5.65	Char 23	2.03	1.88	2.00	1.86
Char 8	2.05	8.67	8.60	6.90	Char 24	1.26	1.29	1.05	1.29
Char 9	1.16	2.08	2.00	1.96	Char 25	3.00	2.99	3.00	3.00
Char 10	1.53	2.90	2.95	2.55	Char 26	1.89	1.89	1.70	1.92
Char 11	7.53	9.00	9.00	8.84	Char 27	4.72	5.07	4.67	3.96
Char 12	1.00	2.55	3.00	2.80	Char 28	2.50	2.51	2.60	2.55
Char 13	68.16	69.53	68.00	69.73	Char 29	1.05	1.40	1.40	1.31
Char 14	1.03	1.07	1.05	1.04	Char 30	2.18	2.07	2.25	2.20
Char 15	66.99	61.90	68.87	65.19	Char 31	7.74	7.90	8.60	8.08
Char 16	4.36	4.04	4.12	4.29					

**Table 3:** Principal component analysis of DUS test characteristics for 180 mung bean varieties.

Characteristics	Principal Component									
	F1	F2	F3	F4	F5	F6	F7	F8	F9	F10
Char 1	0.149	0.100	0.148	−0.044	−0.041	−0.021	0.029	−0.004	−0.106	−0.102
Char 2	−0.025	0.193	−0.044	−0.066	−0.328	−0.102	0.193	−0.064	0.179	−0.032
Char 3	0.151	−0.069	0.157	0.022	−0.053	0.058	−0.034	0.087	−0.145	0.096
Char 4	0.041	0.093	−0.121	0.006	0.050	0.057	−0.081	−0.197	−0.174	0.609
Char 6	−0.069	−0.077	0.189	0.200	0.090	0.075	−0.011	0.017	0.211	0.026
Char 7	0.096	0.021	0.054	−0.118	0.176	0.196	0.116	−0.004	0.117	0.217
Char 8	0.175	0.055	0.098	0.013	−0.018	−0.019	0.020	0.081	−0.162	−0.057
Char 9	0.163	0.041	0.088	0.003	−0.109	0.109	0.066	0.090	−0.050	−0.093
Char 10	0.164	0.050	0.048	0.047	0.002	−0.047	−0.050	−0.014	−0.062	−0.077
Char 11	0.070	0.088	0.135	0.000	0.063	−0.001	−0.272	−0.319	0.126	0.025
Char 12	0.072	−0.018	0.087	0.123	0.248	0.034	0.254	−0.037	0.412	0.054
Char 13	−0.005	0.194	−0.051	−0.231	−0.191	0.055	0.209	−0.162	0.167	0.006
Char 14	0.012	0.147	−0.133	−0.009	0.256	−0.004	−0.098	−0.012	0.003	−0.430
Char 15	−0.079	0.218	0.172	0.134	0.024	−0.025	−0.032	0.008	−0.019	0.138
Char 16	−0.047	0.197	−0.019	0.134	0.032	−0.041	−0.110	0.348	0.027	−0.052
Char 17	0.052	−0.094	−0.080	−0.050	0.288	−0.159	−0.059	0.197	0.133	0.210
Char 18	0.080	−0.037	−0.270	−0.007	−0.046	−0.026	0.052	0.282	0.163	0.010
Char 20	−0.083	0.235	0.155	0.155	0.068	−0.036	−0.109	0.148	−0.021	0.087
Char 21	0.072	0.116	−0.224	0.233	0.167	−0.053	0.017	−0.126	−0.015	−0.002
Char 22	0.085	0.114	−0.152	0.237	−0.031	−0.024	0.136	0.154	0.160	0.011
Char 23	−0.039	−0.037	−0.068	0.183	0.243	0.129	0.314	−0.285	−0.203	−0.081
Char 24	0.009	0.073	−0.140	0.095	0.002	0.148	−0.398	0.018	−0.125	−0.005
Char 25	−0.008	−0.018	0.002	0.193	−0.079	0.470	0.112	−0.005	−0.049	−0.282
Char 26	−0.028	0.104	0.029	−0.181	0.077	0.249	0.124	0.219	0.204	0.185
Char 27	−0.017	0.159	−0.072	−0.294	0.222	0.169	0.063	−0.142	−0.197	−0.039
Char 28	−0.021	0.037	0.105	0.236	−0.003	−0.201	0.186	−0.161	−0.013	0.058
Char 29	0.016	0.040	0.064	−0.183	0.206	−0.421	0.081	−0.056	−0.003	−0.229
Char 30	−0.056	0.018	0.142	−0.168	0.230	0.140	0.000	0.259	−0.083	−0.100
Char 31	0.036	−0.016	0.006	−0.046	−0.001	0.119	−0.358	−0.262	0.492	−0.142

Note: This table shows the loading values of the 29 DUS test characteristics on the first 10 principal components (F1–F10).

**Table 4:** Comprehensive Evaluation and Ranking of Agronomic Characteristics for 180 Mung Bean Germplasm Accessions.

Number	Score	Rank	Number	Score	Rank	Number	Score	Rank	Number	Score	Rank	Number	Score	Rank
15	0.709	1	63	0.601	37	160	0.574	73	92	0.540	109	125	0.458	145
143	0.673	2	166	0.601	38	55	0.571	74	91	0.540	110	7	0.457	146
104	0.659	3	161	0.601	39	100	0.569	75	130	0.539	111	1	0.456	147
80	0.657	4	156	0.601	40	145	0.568	76	131	0.537	112	109	0.454	148
81	0.657	5	48	0.599	41	28	0.565	77	154	0.536	113	101	0.454	149
61	0.656	6	167	0.598	42	79	0.564	78	74	0.536	114	42	0.449	150
99	0.651	7	155	0.598	43	170	0.564	79	29	0.535	115	72	0.448	151
18	0.651	8	39	0.597	44	136	0.564	80	23	0.532	116	114	0.447	152
98	0.648	9	50	0.596	45	117	0.564	81	86	0.526	117	120	0.443	153
150	0.648	10	173	0.592	46	174	0.562	82	128	0.525	118	153	0.442	154
43	0.645	11	34	0.591	47	176	0.562	83	78	0.520	119	113	0.442	155
56	0.643	12	66	0.591	48	147	0.561	84	11	0.517	120	45	0.439	156
134	0.642	13	16	0.588	49	2	0.559	85	32	0.514	121	168	0.438	157
3	0.640	14	25	0.588	50	157	0.558	86	163	0.513	122	152	0.437	158
44	0.639	15	108	0.588	51	67	0.558	87	33	0.511	123	121	0.436	159
4	0.638	16	84	0.586	52	115	0.558	88	36	0.509	124	179	0.435	160
40	0.634	17	135	0.586	53	22	0.558	89	171	0.506	125	52	0.432	161
51	0.634	18	138	0.585	54	19	0.557	90	24	0.502	126	77	0.431	162
96	0.633	19	140	0.585	55	89	0.556	91	35	0.494	127	9	0.431	163
144	0.630	20	13	0.584	56	26	0.555	92	124	0.493	128	21	0.427	164
137	0.623	21	12	0.583	57	75	0.555	93	27	0.489	129	14	0.425	165
162	0.621	22	129	0.582	58	148	0.555	94	116	0.488	130	112	0.423	166
58	0.621	23	164	0.581	59	10	0.554	95	31	0.482	131	70	0.422	167
62	0.620	24	107	0.580	60	180	0.553	96	20	0.480	132	47	0.417	168
17	0.619	25	141	0.580	61	83	0.553	97	151	0.479	133	126	0.417	169
105	0.618	26	46	0.579	62	6	0.553	98	73	0.478	134	97	0.412	170
65	0.618	27	8	0.579	63	133	0.552	99	71	0.478	135	122	0.407	171
110	0.618	28	64	0.578	64	149	0.551	100	60	0.475	136	85	0.406	172
41	0.618	29	5	0.578	65	158	0.551	101	102	0.469	137	57	0.404	173
93	0.617	30	53	0.576	66	146	0.551	102	142	0.466	138	175	0.394	174
49	0.617	31	94	0.574	67	68	0.548	103	169	0.466	139	159	0.393	175
103	0.617	32	139	0.574	68	118	0.548	104	59	0.465	140	87	0.390	176
106	0.616	33	76	0.574	69	69	0.545	105	178	0.464	141	172	0.384	177
111	0.611	34	30	0.574	70	95	0.542	106	127	0.464	142	132	0.375	178
82	0.607	35	54	0.574	71	177	0.541	107	119	0.463	143	123	0.344	179
90	0.601	36	165	0.574	72	37	0.541	108	38	0.460	144	88	0.318	180

**Table 5:** Correlation analysis between DUS test characteristics and mung bean yield.

Characteristics	Yield	Characteristics	Yield
Char 1	−0.02	Char 17	0.14
Char 2	−0.02	Char 18	0.26 **
Char 3	−0.09	Char 19	/
Char 4	−0.02	Char 20	0.08
Char 5	/	Char 21	0.1
Char 6	−0.03	Char 22	0.11
Char 7	0.26 **	Char 23	0.09
Char 8	0	Char 24	0.1
Char 9	0.01	Char 25	0.1
Char 10	−0.06	Char 26	0.17
Char 11	−0.07	Char 27	0.66 ***
Char 12	0.13	Char 28	−0.04
Char 13	0.13	Char 29	0.02
Char 14	0.08	Char 30	0.1
Char 15	−0.01	Char 31	−0.04
Char 16	0.11		

Note: Values represent Pearson correlation coefficients. ** *p* < 0.01, *** *p* < 0.001; “/” indicates no data or not determined.

**Table 6:** The list details the key characteristics.

Character Code	Characteristic	Method of Observation	Type of Expression	Character Code	Characteristic	Method of Observation	Type of Expression
Char 1	Lower hypocotyl: Anthocyanin staining	VG	QL	Char 17	Plant: Angle between branches and main stem	VG	QN
Char 2	Early flowering season	MG	QN	Char 18	Plants: Number of pods	MS	QN
Char 3	Main stem: Color	VG	PQ	Char 19	Plants: Pod-frying property	VG	QN
Char 4	Main stem: Hairs	VG	QN	Char 20	Main stem: Number of nodes	MS	QN
Char 5	Leaves: Number of small leaves	VG	QN	Char 21	Pod: Length	MS	QN
Char 6	Leaf: Small leaf shape	VG	PN	Char 22	Pod: Number of seeds	MS	QN
Char 7	Leaf: Degree of light green color	VG	QN	Char 23	Pod: Shape	VG	PQ
Char 8	Leaves: The anthocyanins at the base of the leaves show color.	VG	QL	Char 24	Pod: Cross-sectional shape	VG	PQ
Char 9	Petiole: Intensity of anthocyanin coloration	VG	QN	Char 25	Pod: Color	VG	PQ
Char 10	Flower: Color of the corolla	VG	PQ	Char 26	Pod: Color of hairs	VG	PQ
Char 11	Flower: Color of the backbone flap	VG	PQ	Char 27	Seed: Weight per Hundred Seeds	MG	QN
Char 12	Flower: Petals show color	VG	PQ	Char 28	Seed: Shape	VG	PQ
Char 13	mature period	MG	QN	Char 29	Seed: Color of the seed coat	VG	PQ
Char 14	Plants: Growth Habit	MS	PQ	Char 30	Seed: Degree of Greenness	VG	QN
Char 15	Plants: Height	VG	QN	Char 31	Seed: The seed coat is shiny.	VG	QL
Char 16	Plants: Number of branches	MS	QN				

Note: Group visual observation (VG); Single measurements (MS); Group measurements (MG); Qualitative characteristics (QL); Pseudo-qualitative characteristics (PQ); Quantitative characteristics (QN).

## Data Availability

The original contributions presented in this study are included in this article; further inquiries can be directed to the corresponding authors.

## Supplementary Materials

The following supporting information can be downloaded at: https://www.mdpi.com/article/10.3390/plants15060932/s1.

## Abbreviations

The following abbreviations are used in this manuscript:

DUS	Distinctness Uniformity and Stability
PCA	Principal Component Analysis
VG	Group Visual Observation
MS	Single Measurement
MG	Group Measurement
QL	Qualitative Characteristics
PQ	Pseudo-qualitative Characteristics
QN	Quantitative Characteristics
CV	Coefficient of Variation
H′	Shannon–Weaver Diversity Index
