# Supplementary material for: Comprehensive Evaluation of Mung Bean Germplasm Resources Based on DUS Test Characteristics
Source: Plants (Basel). 2026 Mar 18;15(6):932. doi: 10.3390/plants15060932 (PMC13030665; doi:10.3390/plants15060932)
Supplement: Supplementary file 1 [file plants-15-00932-s001.zip › plants-4135747-supplementary.pdf]

Table S1. The germplasm resources of 180 mung bean accessions.

Variety number	Variety name	Variety number	Variety name	Variety number	Variety name
1	Yvlv 10	61	Elv 5	121	VC6089-12
2	Tonglv 8	62	1009-2-5	122	VC6307A
3	Binglv 22	63	Bailv 9	123	VC6089-2
4	Liaolv 13	64	Bailv 10	124	VC6307
5	Yulv 11	65	Tong 1188326	125	VC6370-92
6	Jinlv Mung Bean 8	66	Liaolv 10L708-5	126	VC6365
7	Jinlv Mung Bean11	67	Baolv 200810-1	127	VC6459-3-1-35
8	Meng Ke Lv No.3	68	Baolv 201012-7	128	134132
9	Guilv2	69	Kelv 2	129	Hei You Liang
10	Tonglv 11	70	142-139	130	EMS-treated Line 4-14
11	Wankelv 5	71	122-225	131	FLd08-4
12	Chiheilv100	72	Weilv 11	132	Fen Mung Bean No.5
13	Jilv Kang 50-8	73	Weilv 12	133	Ji Heilv 0506 Fan
14	WL1901	74	Wanlv 2	134	Jilv 14
15	Yingge 2	75	JLPX01	135	Liaolv 12
16	Fen Mung Bean 10	76	JLPX02	136	jlse1
17	Weilv 60032	77	Yu Heilv 3	137	Tonglv 5
18	Zhonglv30	78	Yulv 2	138	Tonglv 6
19	Wanlv 11	79	Zhonglv 5	139	Jilv 23
20	Sulv 17-302	80	Binglv 16	140	Jilv 20
21	Wankelv 4	81	Jilv 9	141	Jilv 19
22	Pinlv 2020-25-10-0	82	Jilv 2	142	Jilv 23
23	Baolv 201622-2	83	Baolv 942-34	143	L3001
24	Baolv 201610-3	84	Jin Mung Bean 6	144	L3001
25	172-017	85	Zaolv 1	145	20L5002
26	Wanlv 10	86	Zhonglv 8	146	Chilv 5
27	Weilv 70535	87	Jilv 8	147	Jilv 20
28	WL1902	88	Weilv 50934	148	Zhonglv 31
29	Jilv 0802 Fan-4-1-4-1-2-1-2	89	Weilv 52500	149	Zhonglv 30
30	Jilv 13	90	Jilv 10	150	Binglv 150626
31	Sulv 17-208	91	Jilv 11	151	Binglv 152005
32	Pinlv 2019-26-8-17	92	0802-4-2-1-2-1	152	Bimglv 19
33	172-032	93	HN1023-7-2	153	Wanlv 5
34	Tonglv 9	94	Tong 111411	154	Fen Mung Bean 8
35	11L638	95	Baolv 201322-3	155	HN267
36	Mengkelv 4	96	Baolv 201323-3	156	Zhenglv 25
37	Jin Mung Bean 12	97	Bailv 13	157	L3009
38	Chihuanglv 101	98	Liaolv PB-02	158	L3019
39	Tonglv 10	99	Liaolv 10L701	159	Zhonglv 27

40	1540-6-2-1	100	Zhanglv 2	160	Zhonglv 26
41	1508-15-4-1	101	Su lv 19-118	161	Binglv 20
42	1508-5-4-1	102	Wanlv 5	162	Zhonglv 28
43	1318-5-2-3	103	Wanlv 6	163	Zhonglv 29
44	1507-33-3-1	104	132-346	164	Binglv 11
45	1540-16-2-1	105	112-285	165	Binglv 150626
46	1416-6-3-3	106	1015-38	166	Binglv 152005
47	1540-12-3-1	107	1111-34	167	Binglv 19
48	1512-2-2-1	108	Yulv 9	168	Binglv 21
49	1114-8-1-1-2	109	Pinlv 2014-129	169	Fen Mung Bean 6
50	1520-5-5-1	110	Pinlv 2014-124	170	Jilv 25
51	1111-3-1-2	111	Wankelv 3	171	Zhenglv 24
52	1511-5-3-1	112	VC6368-46-36-2	172	Chilv 121
53	1116-18-3	113	VC6379-23-11G	173	Fenhei Mung Bean 12
54	1415-3-1-2	114	VC6089-3	174	Hangnong Mung Bean
55	1416-4-1-3	115	VC6389-34-7	175	Hangnong 16
56	Binglv 19	116	VC6370-21-3A	176	Qinglv 8
57	Pinlv 2011-06	117	VC6173C	177	Yangyuan Yingge 430
58	Pinlv 2011-12	118	VC6366-7	178	Qinglv 5
59	Jilv 0816	119	VC6370-21-16	179	Binglv141606
60	Sulv 15-11	120	VC3541B	180	Binglv101538
